# Electrorheological Behavior of Suspensions of Polyimide-Based on the Sodium Salt of 2,5-Diaminobenzenesulfonic Acid

**DOI:** 10.3390/polym12051015

**Published:** 2020-04-29

**Authors:** Nikolay Semenov, Alexander Danilin, Yulia Karnet, Elena Kelbysheva

**Affiliations:** Institute of Applied Mechanics of Russian Academy of Science, 125040 Moscow, Russia; semenov@iam.ras.ru (N.S.); andanilin@yandex.ru (A.D.); iam@iam.ras.ru (Y.K.)

**Keywords:** electrorheological suspensions, viscosity, adaptive materials, elastomers, electric field controlled materials, metal-containing polyimide, salt sulfoacid, polymer

## Abstract

Electrorheological suspensions (ERS) of polyimide particles with organoelement fragments from the sodium salt of 2,5-diaminobenzene sulfonic acid were obtained for the first time. Their rheological and electrorheological characteristics, their dependence on the parameters of deformation, and the intensity of the external electric field were studied. It was found that the ERS of **PI-Na** polyimide particles have a significant electrorheological response. Solid-polyimide materials were previously studied using a scanning electron microscope. The effect of the polyimide concentration on the properties of the solution was studied by spectrophotometry. It was shown that polyimide suspension is a result of increasing intensity as the electric field changes the flow type from Newtonian to pseudoplastic due to polarization of the particles and formation of the chain structures along the power lines of the electric field. The influence of temperature on the change of rheological and electrorheological properties of a polyimide ERS in constant electric fields was investigated.

## 1. Introduction

Electrorheological suspensions (ERS) are materials whose rheological properties, such as viscosity, yield strength, shear modulus, etc. [1], change under the influence of an external, electric field. When an electric field is applied, the ERS switch from a low viscosity and a liquid, flowing state to a solid or viscoplastic state. Typically, these changes are reversible and occur within milliseconds. Therefore, ERSs are very promising from a practical point of view.

Due to their unique properties, ERSs are considered “smart materials” [2], whose properties can be adjusted as needed for various practical tasks. For example, ERSs are used as working bodies in electrically controlled mechanical devices, such as controlled dampers, electromagnetic clutches, various vibration protection devices, etc. [3,4]. A number of applications for ERSs in space technology, biomechanics, biomedicine, etc., are proposed in [5].

ERSs are suspensions of polarized solid particles in a nonconductive oil [6]. In the external electric field, the particles are polarized and form chain structures along electric field lines. ERSs, on the basis of polymers [2,7], and in particular, polyimides [8,9] are among the most frequently studied. Polyimide chains are often easy to polymerize, and various aromatic diamines and dianhydrides of acids are commercially available. For practical purposes, it is important to select such polyimide structures that are highly soluble, nonhydrolyzable, and can be used as cast films. Polyimides have a high degree of aromaticity and enable the efficient formation of charge transfer complexes (CTC) for both intramolecular and intermolecular charge transfers, which are necessary for strong electrorheological responses.

It is important to create a material whose properties can be easily adjusted by entering several functional fragments, which can either by themselves or through further modification can dramatically change the properties of the material. It is known that the presence of functional groups containing fluorine reduces the formation of CTC significantly in polyimides and weakens the charge transfer. Also, the materials based on polyimide polymers with the presence of fluorinated side groups are known to have unusual thermomechanical behavior [10]. Only a few examples of metal-containing polyimides have been reported. Such materials are both difficult to make and to characterize, and their properties are not easy to study [11]. Despite these difficulties, polyimides containing metal ions in the main chain were obtained for the first time in reference [12].

Previously, we obtained polyimides that have in the polymer chain a polar and chemically active sulfo group, which is useful for injecting new atoms or groups [13]. In this paper, a new material containing polyimide with Na salt salt-acid fragments and fluorinated groups in the side chain as the dispersed phase is obtained. It is shown that this material has a powerful electrorheological response, which opens the possibility of creating controlled damping devices based on it.

## 2. Materials and Methods 

Tensor 37 (Bruker) IR spectrometer (Bruker Optik GmbH, Ettlingen, Germany) was used to detect infrared (IR) spectra with the resolution of 2 cm^−1^ in KBr cells. The UV/Vis spectra were recorded with a Specord M40 spectrophotometer (Carl Zeiss Industrielle Messtechnik GmbH, Oberkochen, Germany). NMR ^1^H spectra (internal standard – solvent, from Me_4_Si, DMSO-d_6_ – 2.50 ppm) were recorded with a “Bruker Avance 400” spectrometer (400.13 and 100.61 MHz, respectively, Bruker Optik GmbH, Ettlingen, Germany). Rheostress RS 150 rheoviscosimeter (Thermo Haake GmbH, Karlsruhe, Germany) with a plane-to-plane measuring node was used to measure the electrorheological properties. The scanning electron microscope, JSM-6480hv (JEOL Ltd, Akisima, Japan), was used to examine the polyimide sample. Photos of the samples were taken using the Altami MET 5T optical microscope (Altami, St. Petersburg, Russia). The particle-size distribution was analyzed by dynamic-light scattering on a Nanobrook OMNI-BNDL (Brookhaven Instruments, New York, NY, USA) with a laser wavelength of 637 nm. For the synthesis of polyimides, the following reagents were used: 2,5-diaminobenzene sulfonic acids, 4,4′- (hexafluorisopropylidene)diphthalic anhydride, mesitylene, and dimethylformamide (DMF) (all reagents and solvents: Sigma—Aldrich Chemie GmbH, Munich, Germany), PMS-400 (Penta-92, Moscow, Russia). They are commercially available and were used without additional purification.

## 3. Results and Discussion

### 3.1. Synthesis and Characterization of Polyimide

**Scheme 1:**
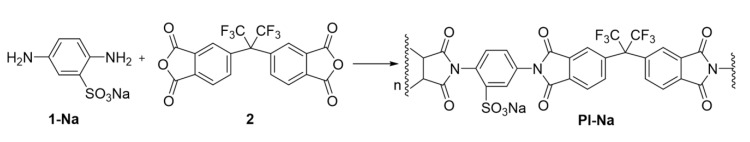
Synthesis of polyimide **PI-Na.**

**PI-Na** polyimide was prepared by a single-stage, high-temperature polycondensation in solution [14] of the sodium salt of 2.5-diaminobenzene sulfonic acid (**1-Na**) and 4.4′-(hexafluorisopropylidene)diphthalic anhydride (**2**) (Scheme 1).

Sodium salt **1-Na** was obtained by the method described in [15]. NaHCO_3_ (2.2 g, 26.6 mmol) was added to a solution of 2.5-diaminobenzene sulfonic acid (5.0 g, 26.6 mmol) in 30 mL of deionized water and 20 mL of acetone in portions. The solution was heated until the water was completely removed, and the remaining substance was vacuum-dried at 90 °C for 4 h. The yield was of **1-Na** 5.5 g (98%). NMR ^1^H was (400 MHz, 25 °C, DMSO-d_6_): 4.24 (s, 2H, NH_2_), 4.80 (s, 2H, NH_2_), 6.36 (m, 2H, H-Ph), and 6.84 (br.s., 1H, H-Ph). IR spectrum (KBr) and ν/cm^−1^: 3408, 3383, 3322 ν(NH), 1182, and 1028 ν(SO).

Predictability of the impurity composition, a high degree of imidization, and the thermal and chemical stability of the polymer are the main requirements to a method for making polymer material. 

X-ray fluorescence elemental analysis data confirmed the elemental composition of the resulting polyamide (Table 1).

**Figure 1:**
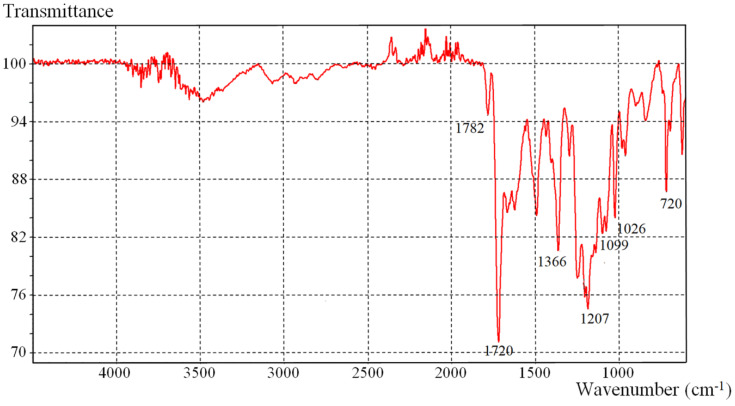
Infrared (IR) spectra of **PI-Na**.

The IR spectrum shown in Figure 1 confirms the structure of Na-containing polyimide **PI-Na.** The IR data exhibits characteristic-absorption bands corresponding to the deformation oscillation of the image ring (C–N) located in the region of 720 cm^−1^, as well as the peaks at 1782 and 1720 cm^−1^, which correspond to symmetrical and asymmetric oscillations of the imide carbonyl groups (N–C=O). A peak in the area of 1026 cm^−1^ and a sharp peak in the area of 1099 cm^−1^ are assigned to the SO_2_ group. The bands in the area of 1366 and 1207 cm^−1^ correspond to the vibrations of the C–F group. It should be noted that the polyimide **PI-Na** is quite hygroscopic and requires predrying before the instrumental analysis.

Analysis of solution **PI-Na** by dynamic-light scattering shows that the studied polymers are polydisperse systems. The size of the studied particles in the solution mainly lies in the range from 100 to 295 nm in DMF.

**Figure 2:**
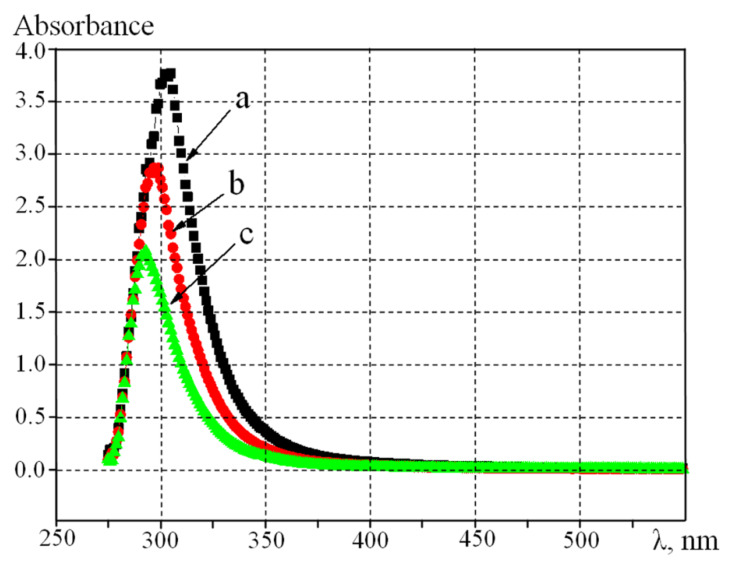
UV-visible spectra of polyimide solutions: (**a**) 1%, (**b**) 0.5%, and (**c**) 0.25% in dimethylformamide (DMF).

UV-visible spectra (Figure 2) of polyimide solutions reveal the influence of the solution concentration on their properties. As shown in Figure 2, reducing the concentration of the solution by four times leads to a hypsochromic (or blue) shift from 306 to 290 nm. Likely, this is due to the presence of intermolecular interactions between segments of polyimides in the solution. The higher the concentration of the solution, the smaller the distance between the segments, and the interaction between them increases. This is consistent with the literature data in [16].

**Figure 3:**
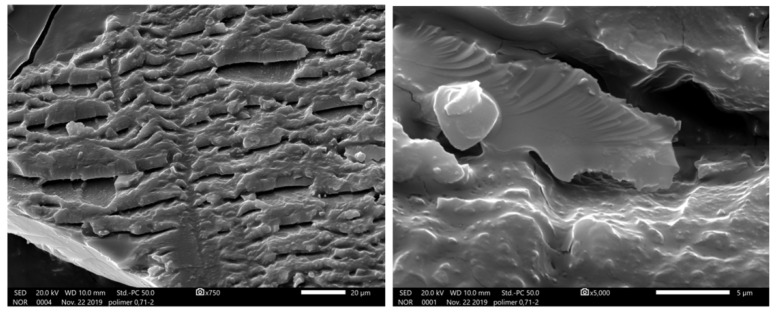
Electron-microscope images taken at different locations of the polyimide-material surface: center, 750× (**left**) and edge, 5000× (**right**).

The surface morphology of the material was studied with electron microscopy. These images (Figure 3) show that the polyimide is a large crystallite with a nonuniform layered structure. The results of X-ray diffraction analysis of the surface are shown in Table 2.

### 3.2. Electrorheological Experiments

Previously, we studied the electrorheological properties of materials based on polyimide particles of different structures, such as PMDA-APS and BTDA-ODA or BTDA-APB [17]. It was shown that polyimides with an articulated bond of polymer’s chain exhibit a large ER effect. Furthermore, polyimide particles with some structure exhibited a negative ER effect, e.g., on BTDA-APB. This was an effect where the viscosity drops while the electric field strength increases. This was also be observed using optical microscopy. When applying an electric voltage to ER, polyimide particles showing negative electrorheological effects did not move towards power lines and did not create structured chains, but started rotating around their axis with precession [17] or separating on the cathode. Entering the polar-sulfonic group [13] into a polyimide structure allowed creating polymers without articulated bonds with a direct ER effect. It also opened the extended possibilities for functional modifications. Specifically, the creation of organometallic complexes and salts. New materials began to show a direct ER effect under an applied electrical field due to polyimide particles moving towards the power lines of the electric field strength. We suggested that protons in SO_3_H are mobile and are distributed between acid centers; thus, creating a partial charge in the molecule. This, in turn, led to a dramatic change in the mechanical properties of electrorheological suspensions. At the same time, the sulfo group was useful for additional adjustments of required properties in new materials. In this work, the electrorheological properties of polyimides with modified sulfonic acid fragments were firstly obtained and studied.

**Figure 4:**
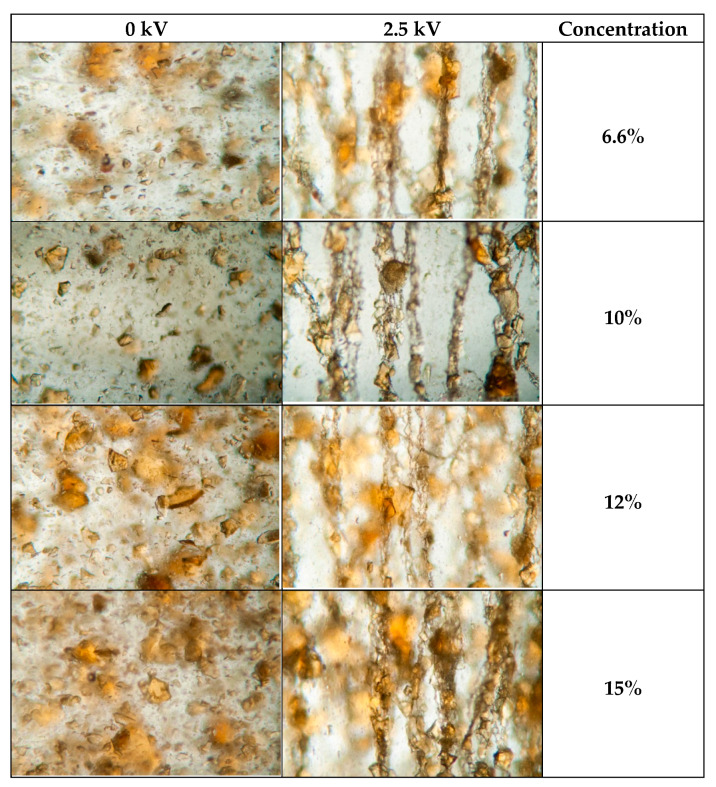
Micrographs of suspensions of polyimides without a field (**left**) and with a field of 2.5 kV (**right**) (scale 1 mm by the height of the picture).

Microscopic photo images of suspensions with different concentrations, with and without the presence of the electric field, are shown in Figure 4.

Figure 4 shows that the **PI-Na** suspension particles in the absence of an electric field are in the form of unevenly dispersed suspensions, with solid-phase particles of different sizes. After applying the electric field, the polyimide particles line up along the field lines of the electric field, forming thread-like structures. There are always four such structures per millimeter of the analyzed length in the transverse direction to the electric field, regardless of the concentration.

#### 3.2.1. Electrorheology Characteristics

In this study, electrorheological tests were conducted on the plane–plane operating unit. An ER unit is a system of flat, stainless steel electrodes (working surface diameter 35 mm) isolated from the onboard viscometer systems by a ceramic axis. The top electrode (a flat disc) was fixed on an axle connected to the step drive that generates different speeds of rotation (revolutions). The second electrode was also disk-shaped, immobile, and was located under the first. The mutual position of work surfaces was rigidly fixed with a special structure. To ensure a uniform clearance between the flat surfaces (1 mm), the distance between them was carefully calibrated. The voltage applied to the cell was fed via a slider contact to the bearing, which was connected to the metal axle of the upper electrode and the lower fixed electrode. Electrorheological experiments were performed in the mode of a steady shear flow, to which the electric field was applied in the normal direction in the voltage range from 0 to 4 kV. Shear velocity change interval in these studies ranged from 0.1 to 200 and 30 s^−1^ in the experiment with constant stress. The temperature was controlled by a liquid-thermos controller with 0.1 °C accuracy on the low plate.

**Figure 5:**
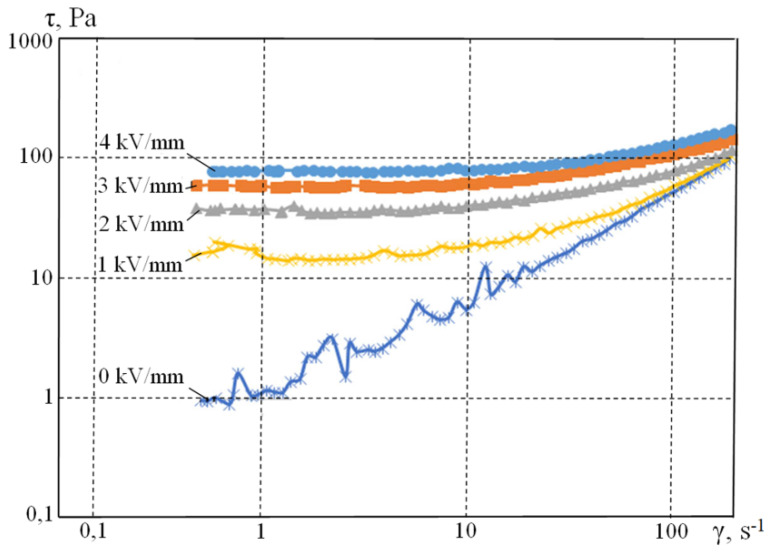
The dependence of stress versus the shear rate of the suspension **PI-Na**.

**Figure 6:**
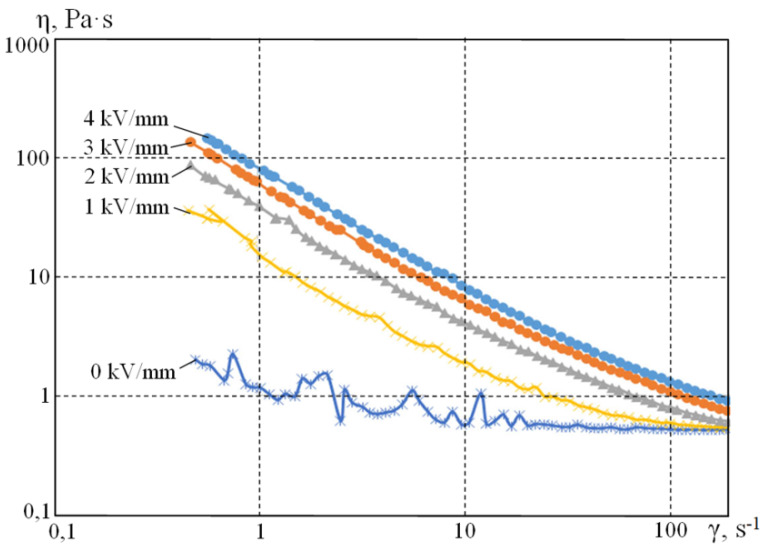
The dependence of viscosity on the shear rate of the suspension **PI-Na**.

Rheological measurements were performed with a gradually increasing shear-strain rate from 0.1 to 200 s^−1^ at the temperature of 20 °C for 10% by weight of **PI-Na** polyimide suspensions. The results are shown in Figure 5 and Figure 6.

Figure 4 and Figure 5 show that the electric field changes the suspension-flow pattern from linear (Newtonian) to viscous (Ostwald–de Ville). For a given shear rate, the viscosity of the suspension increases with the increase in the applied electric voltage, which is typical for electrorheological suspensions. With the increase in the shear rate, the structural bonds of the filler do not have enough time to form, and the value of viscosity converges to that of without the field.

#### 3.2.2. The Influence of Concentration

Suspensions with solid-phase concentrations of 6.6%, 10%, 12%, and 15% by weight were investigated. As the increase in the concentration of solid particles leads to the increase of initial viscosity of the suspension, a direct comparison of the flow curves is not suitable for comparison of the effect of the applied electric field at different concentrations. The relative parameter of the given value of tangent stresses: $\Delta \tau /\tau_{0}=\left(\tau -\tau_{0}\right)/\tau_{0}$ is more appropriate. Here τ is the tangent stress, and $\tau_{0}$ is the tangent stress at zero electric field strength.

**Figure 7:**
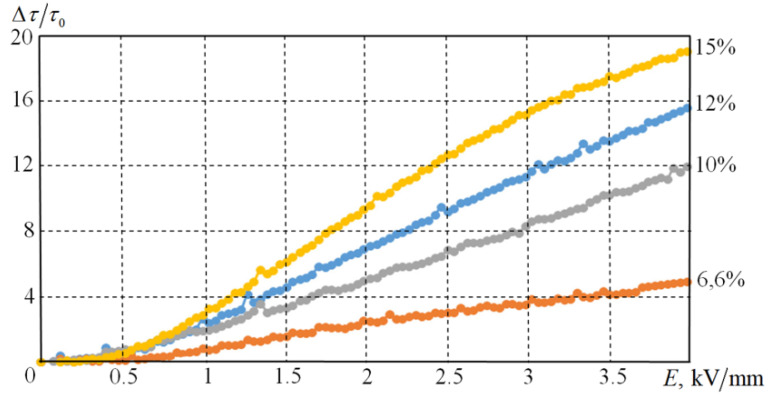
The dependence of the adjusted value of shear stresses on the electric field at 20 °C.

Figure 7 shows the values of the shear stresses measured at a constant shear rate of 30 c^−1^ and a temperature of 20 °C as a function of voltage, which varied from 0 to 4 kV/mm.

Our results clearly demonstrate that the increase in concentration leads to an increase in the ER effect. For the suspension with the 6.6% of particles by mass, the tangential stresses show a four-fold increase at 4 kV / mm, and a nearly 20-fold increase for the 15% suspension.

#### 3.2.3. The Influence of Temperature

**Figure 8:**
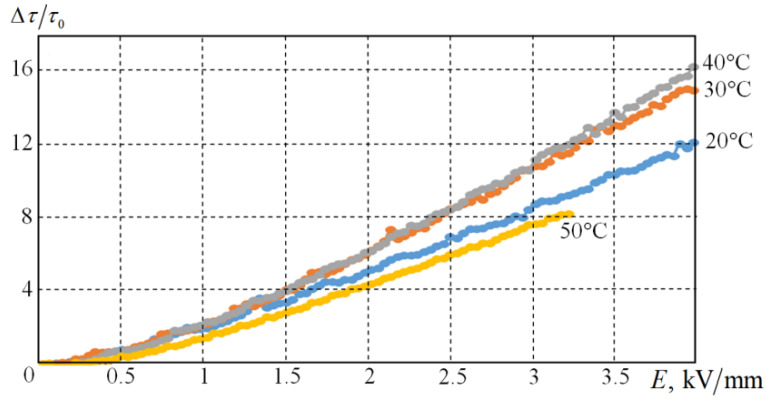
The dependence of the adjusted value of shear stresses on the electric field in temperatures 20, 30, 40, and 50 °C for suspensions of concentration of polyimide **PI-Na** 10 wt %.

Figure 8 shows shear stress at a constant shear rate as a function of the applied voltage in the range between 0 and 4kV for several temperatures between 20 and 50 °C. 

At 50 °C and the voltage of ~3 kV / mm, a short circuit occurs in the measuring node, and the current increases abruptly. Apparently, this is due to the presence of sodium ions, which leads to the appearance conductivity in the suspension.

**Figure 9:**
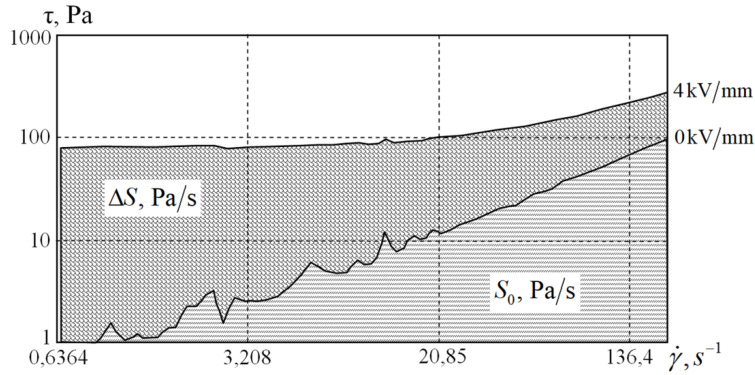
The difference of area under a flow curve for a 10 wt % suspension of polyimide **PI-Na** at 20 °C and 0 and 4 kV.

As the viscosity of the medium decreases with an increase in temperature, a direct comparison of flow curves is inconvenient for evaluating the effect of temperature. A comparison of the area between the flow curves is much more informative. It is well known that the area under the flow curves represents the energy (W/m^3^) required for deforming a single volume of the sample [18,19]. The area between the curves represents the energy that is required to destroy the filaments of the filler. Therefore, comparing the areas under the flow curves at different temperatures is convenient for representing the contribution of oriented chains to the changes in rheological properties. Let us denote the areas under the flow curves in the absence of voltage and in its presence by $S_{0}$ and $S_{i}$, respectively. Let us also introduce the value of the area difference $\Delta S=S_{i}-S_{0}$, as shown in Figure 9 at 20 °C and 4 kV. Then the ratio ∆*S*/*S*_0_ shows how many times more or less energy needs to be spent on the deformation of the sample in the applied electric field compared to the deformation process without a field.

**Figure 10:**
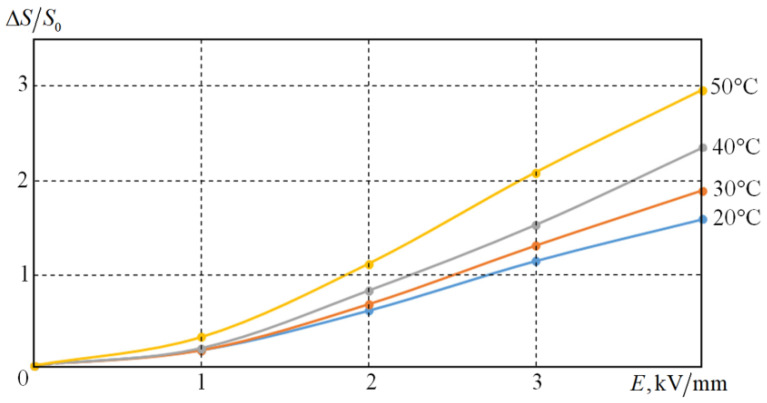
The dependence of ∆S on the electric field in temperatures 20, 30, 40, and 50 °C.

Figure 10 shows the dependence of ∆*S*/*S*_0_ versus the electric field strength. It can be seen that when the temperature increases, the relative energy cost for the deformation of the sample increases. This is because the viscosity of the medium decreases with temperature, the forces of interaction between the electrorheological particles remain the same.

## 4. Conclusions

In this work, suspensions of polyimide particles with metal-containing fragments were obtained for the first time. Their rheological and electrorheological characteristics, their dependence on the parameters of deformation, and the intensity of the external electric field were studied. It was found that the ERS of **PI-Na** polyimide particles has a significant electrorheological response. It was shown that increasing the concentration of filler particles in the suspension leads to an increase in the ER effect. Thus, at 6.6% by mass of particles in the suspension by mass, shear stresses increased four-fold at 4 kV/mm, and at 15%, the 20-fold increase was observed. The influence of temperature changes on the rheological and electrorheological properties of polyimide ERSs in constant electric fields was demonstrated. It was shown that as the temperature increased, the viscosity of the medium fell, but the structure of the sample did not change. Thus, the change in electrorheological properties and electrorheological response can be most efficiently achieved by the simultaneous effect of the electric field and temperature. Thus, our data should be taken into account for designing devices that use ERSs in conditions when they are subject to heating during operation.

## Figures and Tables

**Table 1:** Thermal properties and elemental analysis data of the **PI-Na**.

Polyimide	Formula	*T*_g_ (°C)	Elemental Analysis (%)						
**PI-Na**	C_25_H_9_O_7_F_6_NaN_2_S	420		**C**	**H**	**N**	**F**	**S**	**Na**
			Calcd	48.56	1.47	4.53	18.44	5.18	3.71
			Found	47.99	2.27	5.13	17.01	4.36	3.50

**Table 2:** Data quantitative X-ray microanalysis for the surface of PI-Na-based material obtained using REM.

Polyimide	Formula	Analysis (%)					
**PI-Na**	C_25_H_9_O_7_F_6_NaN_2_S	**C**	**O**	**N**	**F**	**S**	**Na**
		39.27	17.70	8.61	21.06	7.49	5.88
